# Cross-Modal Re-Organization in Clinical Populations with Hearing Loss

**DOI:** 10.3390/brainsci6010004

**Published:** 2016-01-26

**Authors:** Anu Sharma, Hannah Glick

**Affiliations:** Department of Speech, Language & Hearing Science, Institute of Cognitive Science, University of Colorado, 2501 Kittredge Loop Road 409 UCB, Boulder, CO 80309, USA; hannah.glick@colorado.edu

**Keywords:** cross-modal re-organization, sensitive period, auditory cortex, auditory deprivation, auditory evoked potentials, visual evoked potentials, somatosensory evoked potentials, P1 CAEP, cochlear implants, single-sided deafness, age-related hearing loss

## Abstract

We review evidence for cross-modal cortical re-organization in clinical populations with hearing loss. Cross-modal plasticity refers to the ability for an intact sensory modality (e.g., vision or somatosensation) to recruit cortical brain regions from a deprived sensory modality (e.g., audition) to carry out sensory processing. We describe evidence for cross-modal changes in hearing loss across the age-spectrum and across different degrees of hearing impairment, including children with profound, bilateral deafness with cochlear implants, single-sided deafness before and after cochlear implantation, and adults with early-stage, mild-moderate, age-related hearing loss. Understanding cross-modal plasticity in the context of auditory deprivation, and the potential for reversal of these changes following intervention, may be vital in directing intervention and rehabilitation options for clinical populations with hearing loss.

## 1. Introduction

Brain plasticity is an often overlooked yet important factor that may influence clinical outcomes in hearing impaired individuals who receive intervention via hearing aids or cochlear implants. Moreover, brain plasticity provides the framework upon which rehabilitation and therapy initiatives for these clinical populations could be based. Plasticity, in its broadest form, refers to the brain’s ability to change, for neurons and networks to alter their function as a result of intrinsically or extrinsically driven factors. Here, we review brain plasticity in hearing loss, including experience-driven plasticity, cross-modal, and intra-modal plasticity in early development and adulthood. Using electroencephalographic (EEG) techniques, we provide evidence of cross-modal re-organization in patients with hearing loss across the age spectrum and discuss its possible clinical implications.

## 2. Developmental Plasticity in Hearing Loss

During development, both intrinsic and extrinsic factors have a profound influence on cortical maturation of the auditory system. In infants and young children, the cortical auditory evoked potential (CAEP) response can be used as a biomarker in assessment of the development of the central auditory pathways. For normal hearing infants and young children, the morphology of the CAEP response is dominated by a single obligatory, positive peak known as the P1 component. In early childhood, the P1 CAEP response occurs around 200 and 300 milliseconds. During development, an intrinsically regulated period of synaptogenesis occurs in auditory cortex, the peak of which occurs between 3.5 and 4 years of age, and after which extrinsically driven factors such as sensory stimulation cause refinement and pruning of central auditory pathways [1,2]. As a child’s auditory system becomes refined by intrinsic and extrinsic inputs, the P1 CAEP response decreases rapidly early in childhood and then gradually into adulthood, eventually reaching a peak latency of 50 to 70 milliseconds. This decrease in P1 latency reflects refinement in the efficiency of sound transmission along the central auditory pathways to the auditory cortex. During pre-adolescence, a change in morphology of the CAEP waveform is also observable, where we see the emergence of later CAEP components, known as the N1 and P2. Unlike the P1 component, with cortical sources in primary auditory cortex and the thalamus, the N1 and P2 components reflect higher order auditory processing, with cortical sources in primary as well as secondary auditory cortex [1,2,3,4,5,6].

The period of synaptogenesis early in development coincides with a critical or sensitive period for cortical auditory development in children, in which the central auditory pathways are maximally plastic. During this sensitive period, extrinsic sensory stimulation has the ability to profoundly shape the development and organization of the auditory cortex [4,7,8,9,10,11,12]. For example, in a study by our group [4,7,8,13] CAEP responses were recorded in 245 bilateral congenitally deaf children who received cochlear implants. These children were divided into three groups: early implanted children who received an implant before the age of 3.5 years, children implanted between the ages of 3.5 and 7 years, and late-implanted children who were implanted after the age of seven years. Their P1 responses were compared to 95% confidence intervals of P1 latency established in a large population of typically developing, normal hearing children at various ages [7]. Results from this study indicate that early implanted children—receiving their cochlear implant before age 3.5 years—demonstrate a P1 response that falls within normal limits compared to typically developing, age-matched normal hearing children. In contrast, congenitally deaf children receiving an implant after the age of seven years exhibited abnormal P1 responses with latencies falling outside of the 95% confidence limits for P1 latencies in typically developing, normal hearing children. Children implanted between 3.5 and 7 years demonstrated variable responses, with roughly half of the children in this group demonstrating a P1 response within normal limits. Later studies by Sharma and colleagues further documented this relationship by examining the developmental trajectory of the P1 response in cochlear-implanted children over time [7]. Sharma and colleagues reported that P1 latency decreased rapidly and eventually fell within age-appropriate limits within six to eight months of cochlear implant use in the group of early cochlear-implanted children, whereas children who were late implanted showed only a modest decrease in P1 latency and developmental trajectories never falling within normal age limits even after years of implant use [7]. These findings are supported by animal studies, in which a sensitive period for cortical auditory development has been documented [1,14,15]. Evidence of the sensitive period based on CAEP P1 responses are further supported by behavioral studies demonstrating that audiological intervention (e.g., cochlear implantation) within the sensitive period yields the most optimal outcomes for children with bilateral deafness who receive cochlear implant [16,17,18,19].

The N1 component of the CAEP response also demonstrates clinical utility as a biomarker in assessing cortical auditory development in children with hearing loss. Unlike the positive P1 component which arises from auditory cortex, cortical sources of the N1 arise from higher-order auditory cortex and cortico-cortico coupling—processes that facilitate higher-order auditory and language processing [20,21]. In typically developing, normal hearing children, the N1 component emerges as early as age three years and can be reliably observed by age seven years [22]. In fact, those children who are early-implanted under the age of three years show typical N1 patterns when compared to the normal hearing children (71% of these early-implanted children develop an N1 response between ages six to nine and in 100% of these children between ages 9–12) [23]. In contrast, children who are late-implanted rarely ever develop an N1 response [20,22,23]. As a whole, the N1 biomarker may provide useful information regarding age-appropriate higher-order auditory cortical development. This tool can be used in conjunction with the P1 biomarker, together providing clinically useful information regarding brain development and maturation in primary and higher-order auditory cortices [4,23,24,25].

Taken together, the aforementioned studies provide important validation for the idea of early implantation in bilateral deaf children. However, what these studies do not explain is the reason why late-implanted children rarely develop normal speech and language skills. One theory is that auditory deprivation may affect cortical connectivity, and that alterations in this connectivity may consequently affect normal development of speech and language abilities [2]. Animal research suggests that laminar and cortico-cortico function are affected by auditory deprivation extending beyond the sensitive period [14]. However, if hearing is restored within the sensitive period, there exists the potential to reverse some of these changes [26]. Feed-forward and feedback projections between the cortex and the thalamus and cortico-cortico connectivity are vital in the modulation of incoming auditory stimuli and other forms of complex auditory processing. If the connectivity between cortico-cortico networks is disturbed (e.g., if coupling between primary auditory cortex and secondary auditory cortex and association cortices is disrupted), then this could affect language learning during development. Poor speech and language outcomes and lack of an N1 CAEP component in late-implanted children are thus suggestive of thalamo-cortical and cortico-cortico deficits [2,22]. Ultimately, these changes in connectivity may leave auditory cortex, and in particular higher auditory cortical regions, vulnerable to recruitment from other forms of sensory processing, including vision and somatosensation [5,27,28,29]. This is a process known as cross-modal cortical re-organization.

## 3. Cross-Modal Plasticity in Cochlear Implanted Children

Cross-modal cortical re-organization is a form of cortical plasticity. When cortical regions do not receive adequate sensory input (e.g., auditory cortices as in deafness), these brain regions become vulnerable to recruitment from other sensory modalities like vision, which may repurpose auditory areas for visual processing [30]. For example, enhanced visual motion detection in congenitally deaf cats is subserved by the posterior auditory field, indicative of cross-modal re-organization of auditory cortex [30]. Similarly, behavioral evidence of enhanced visual abilities have been documented in humans [31], as well as in fMRI studies in which visual motion stimuli elicits activation of superior temporal gyrus in deaf adults [32]. Changes in functional connectivity in deaf adults further supports these findings, with increased functional connectivity observed in superior temporal gyrus in deaf compared to normal hearing adults adults indicating compensatory modification in cross-modal cortico-cortico connections in deafness [32].

There is also clear evidence of cross-modal re-organization during development, for example, in children with cochlear implants. Lee and colleagues have conducted several studies in which speech perception outcomes following cochlear implantation are positively correlated with pre-implant resting hypometabolism in auditory cortex measured via fluorodeoxyglucose positron emission tomography (FDG-PET) [11,33,34]. This measure of glucose metabolism is an indirect measure of cross-modal re-organization by auditory cortex, whereby higher metabolic rates observed in late-implanted children are suggestive of cross-modal recruitment of auditory cortex for sensory processing. Giraud and Lee (2007) and Lee *et al.* (2007) found that among cochlear implanted children, those that had good speech and language outcomes recruited dorsal networks including dorsolateral pre-frontal cortex, while those that had poor clinical outcomes recruited ventral networks showing high metabolic activity in ventral visual regions [33,35]. Significant sources in ventral visual regions in the cochlear implanted children is suggestive of cross-modal re-organization, and these cross-modal changes were correlated with poorer speech perception and language outcomes in this group of children [33,35].

**Figure 1:**
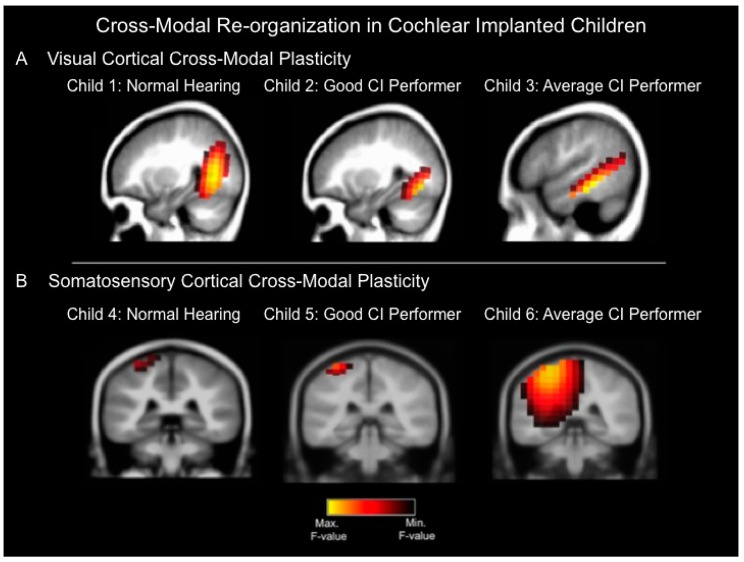
(**A**) Current density reconstructions for the cortical visual evoked potential (CVEP) P2 component in three different children: a child with normal hearing (Child 1), a cochlear implanted (CI) child who is a good performer with good speech perception (Child 2), and a cochlear implanted child who is an average performer with an average speech perception score (Child 3). The visual stimulus consisted of a visual motion stimulus. Yellow regions demonstrate the localization of cortical sources with higher statistical likelihood, whereas darker regions reflect cortical sources with lower statistical likelihood. The normal hearing child (Child 1) and good CI user (Child 2) show significant sources only in expected higher-order visual processing regions in response to the visual motion stimulus, whereas the average CI user (Child 3) demonstrates evidence of cross-modal plasticity where the visual motion stimulus results in significant cortical sources in higher-order visual as well as temporal (auditory) cortical areas; (**B**) Current density reconstructions for the cortical somatosensory evoked potential (CSSEP) N70 component in 3 children: A child with normal hearing (Child 4), a cochlear implanted child with a good speech perception score (Child 5), and a cochlear implanted child with an average speech perception score (Child 6). Vibrotactile stimulation of the right index finger results in significant cortical sources only in somatosensory cortex in the normal hearing child (Child 4) and good CI user (Child 5), whereas the average CI user (Child 6) demonstrates significant sources in somatosensory cortex, as well as temporal (auditory) cortical regions, indicative of cross-modal re-organization [23].

While the aforementioned studies suggest cross-modal plasticity in groups of children, in a recent investigation [23], we examined whether we might see evidence of cross-modal processes in individual children and whether they might be related to clinical outcomes with the cochlear implant. We recorded cortical visual evoked potentials (CVEPs) and cortical somatosensory evoked potentials (CSSEPs) in six different children with normal hearing and with cochlear implants in addition to assessing speech perception using clinical tests [23,36]. We used the standardized Low-Resolution Brain Electromagnetic Tomography (sLORETA) to localize electric neuronal source distribution (standardized current density reconstruction) in the cortex using a standardized head model [37]. sLORETA estimates statistical maps with low error of the location of underlying current sources on the scalp for each component in the event related potential [37]. Figure 1A depicts current density reconstructions for the CVEP P2 component for three children: A 10 year-old child with normal hearing (left panel, child 1), an eight year-old cochlear implanted child with a speech perception score of 96% on the Lexical Neighborhood Test (middle panel, child 2), and a seven year-old cochlear implanted child with a speech perception score of 67% on the Multisyllabic Lexical Neighborhood Test (right panel, child 3). In response to visual stimulation, both the normal hearing (left panel, child 1) and good cochlear implanted (CI) performer (middle panel, child 2) show significant cortical sources in higher-order occipital cortices such as middle occipital gyrus, fusiform gyrus, and lingual gyrus. In contrast, the average CI performer (right panel, child 3) shows cortical sources in occipital areas, as well as auditory cortical regions (superior temporal gyrus and medial temporal gyrus), indicative of cross-modal recruitment of auditory (temporal) regions for visual processing. Figure 1B depicts current density reconstruction (CDR) for 3 the CSSEP N70 component computed for three different children: A normal hearing seven-year old child (left panel, child 4), a 13-year old cochlear implanted child with a speech perception score of 94% on the Consonant Nucleus Consonant (CNC) test (middle panel, child 5), and a 15 year-old cochlear implanted child with average performance of 76% on the CNC speech perception test (right panel, child 6). In response to vibrotactile stimulation, both the normal hearing child (left panel, child 4) and good CI user (middle panel, child 5) show significant sources in somatosensory cortex including post-central gyrus. In contrast, the 15 year-old average CI user (right panel, child 6) shows cortical sources in somatosensory cortex as well as auditory (temporal) cortical regions including superior and transverse temporal gyrus and parietal cortex, suggesting cross-modal recruitment of auditory (temporal) regions for somatosensory processing [23].

Though the data presented above represent case study evidence and should accordingly be interpreted as such, these findings add to the growing body of evidence that visual and somatosensory cross-modal re-organization does occur in some cochlear implant recipients, and that cross-modal recruitment may be related to speech perception outcomes [33,38,39,40]. With future research, brain markers of cross-modal plasticity may become useful in exploring potential causes for the variability in speech perception outcomes in patients with hearing loss receiving audiological intervention. In the future, greater understanding of cross-modal sensory re-organization in children with hearing loss may provide a valuable tool in predicting success with a cochlear implant or hearing aid. Knowledge of compensatory mechanisms may help interventionists individualize rehabilitation and training programs. It is possible that multimodal approaches to rehabilitation following cochlear implantation or other forms of audiological intervention may be beneficial for some patients. For example, in a series of studies by Strelnikov, cochlear implanted adults showed more benefit from auditory-visual cues compared to adults with normal hearing [38]. Further, the functional level of the visual modality in deaf adults was related to the auditory recovery, six months after cochlear implantation, suggesting that auditory-visual synergy may facilitate better speech perception abilities following cochlear implantation [38,39]. In animal models, there is also some evidence to support the use of multisensory training to improve sound localization following bilateral cochlear implantation [40]. From a clinical perspective, research in this area may lead to the development of more targeted and effective rehabilitation programs for adults and children with hearing impairment who receive audiological intervention, and may help the clinician select individualize rehabilitation programs for different patients.

## 4. Can Cross-Modal Plasticity be Reversed after Clinical Intervention?

As the criteria for cochlear implantation in children expands, clinical evidence of cross-modal plasticity may play a critical role in determining whether implantation may be beneficial in non-typical cases of pediatric deafness. For example, cochlear implantation for cases of single-sided deafness (SSD) is not currently approved by the Federal Drug Administration (FDA) in the United States. Research demonstrating the efficacy of cochlear implantation in both adults and children with SSD is limited to behavioral evidence, though emerging data suggests increased speech perception abilities in quiet and in noise, as well as improvement in binaural hearing in some patients with SSD following cochlear implantation [40,41,42,43,44]. Sharma and colleagues (2015) recently documented cortical maturation of auditory cortex in a child with SSD following cochlear implantation, as well as changes in cross-modal cortical re-organization [45]. The child passed her newborn hearing screening but was identified with a moderate hearing loss in her right ear at age five years, and this hearing loss progressed to severe-profound by the time she reached age nine years. At age 9.86 years, the child received a cochlear implant in her right ear. Cortical visual evoked potentials (CVEPs), cortical somatosensory evoked potentials (CSSEPs) and cortical auditory evoked potentials (CAEPs) were recorded in this child prior to cochlear implantation and at subsequent intervals post-implantation using 128-channel, high density EEG. Current density source localization and behavioral correlates of speech perception in noise and sound localization were also measured (see [45] for details about stimuli and methodology used). Pre-CI, the visual motion stimulus elicited significant cortical sources in left frontal gyrus, inferior temporal gyrus, and medial temporal gyrus, consistent with cross-modal recruitment of auditory (temporal) areas for visual processing. Post-CI, in contrast, cortical sources were observed in expected visual motion processing regions including fusiform gyrus, as well as auditory processing regions such as left superior and middle temporal gyrus. In this case, cross-modal re-organization by vision reversed partially following cochlear implantation. In contrast, before implantation, vibrotactile stimulation of the right index finger elicited sources in somatosensory cortex including pre- and post-central gyrus, inferior and superior parietal lobules), as well as temporal (auditory) cortical regions including middle temporal gyrus and the insula, suggesting cross-modal recruitment of auditory regions for somatosensory processing. Post-CI, however, in response to the same stimulus, we saw significant cortical sources only in somatosensory regions (pre- and post-central gyrus, inferior and superior parietal lobule), demonstrating a complete reversal of somatosensory cross-modal plasticity following implantation [45]. Given that this child showed excellent speech perception and localization with her implant, we may conclude that the reversal of cross-modal plasticity after implantation was positively correlated with the ability to derive maximal benefit from the cochlear implant.

While only a singular case, this child with SSD exhibited a high degree of neuroplasticity well-beyond the sensitive period for bilateral deafness, including partial reversal of cross-modal recruitment of auditory cortex by vision and complete reversal of cross-modal recruitment of auditory cortex by the somatosensory system following implantation, and the development of an age-appropriate CAEP response after implantation [45]. Future research should examine the different trajectories for visual *vs.* somatosensory cross-modal plasticity in hearing loss. For example, it’s possible that persistence of visual cross-modal reorganization is maintained by the functional role that vision plays in everyday communication, consistent with previous reports that visual intra-modal plasticity may be beneficial for audiovisual speech perception in cochlear implantation ([45]; also see below discussion on intra-modal plasticity). On the other hand, somatosensory cross-modal plasticity may not serve a similar functional purpose in humans, but may underlie the proximity and similarity of the auditory and somatosensory systems or may correlate with speech production which incorporates vibrotactile cues. Evidence for somatosensory cross-modal plasticity in deafness has been described in animal literature. For example, Meredith and colleagues (2012) demonstrated that adult ferrets show a substantial increase in the proportion of neurons in auditory cortex (anterior auditory field) that respond to somatosensory stimuli following ototoxic-induced moderate hearing loss [46]. While this finding contributes to one of the mechanistic theories of cross-modal plasticity whereby sensory deprivation in one modality leads to excitatory/inhibitory imbalances system-wide, future research is needed to better understand the functional consequences of somatosensory cross-modal plasticity in humans and its functional consequences.

## 5. Cross-Modal Plasticity in Cochlear Implanted Adults

The phenomenon of cross-modal recruitment of auditory processing areas by vision has also been reported in profoundly deaf adults with and without cochlear implants [28,39,47,48,49,50,51]. Cross-modal plasticity may be adaptive or maladaptive in nature. In the case of auditory deprivation due to hearing loss, adaptive brain plasticity may allow an individual to make use of their hearing aid or cochlear implant, to learn oral speech and language, to improve auditory perception in everyday listening environments. Maladaptive plasticity, in contrast, may prevent an individual from making expected strides in auditory perception and speech comprehension. Whether cortical re-organization in hearing loss is adaptive or maladaptive in nature is not so straightforward. For example, visual cross-modal recruitment of auditory cortical areas by vision in deafness may be adaptive in the sense that it may allow an individual to cope in difficult listening situations, relying more on facial visual cues to enhance speech perception, but it may also be maladaptive in the sense that cross-modal re-organization may fundamentally alter allocation of those auditory cortical regions for auditory processing, possibly inhibiting an individual from maximizing use of their hearing aid or cochlear implant [36,52]. In general, cross-modal re-organization seems to be more pronounced in cochlear implanted adults with poor and moderate speech perception performance, as opposed to good performers [46,49,53,54]. For instance, Doucet and colleagues (2006) assessed high density EEG in a group of adults with cochlear implants who were good and poor users. Their results suggest that poor users show more cross-modal re-organization (demonstrating cortical sources over visual, auditory, and multisensory cortical areas in response to visual stimulation), while good users demonstrate little cross-modal reorganization (cortical sources restricted to visual processing areas in response to visual stimulation) [47]. Similarly, Buckley and Tobey (2011) found increased cortical visual evoked potential response amplitude over the right temporal lobe in pre-lingually deafened adult cochlear implant users, suggestive of cross-modal plasticity, and these changes were associated with poorer speech perception [48]. Another study by Sandmann and colleagues (2012) report reduced visual evoked potential (P100) amplitude, reduction in source activity over visual cortex, and significant sources in right auditory cortex in a group of cochlear implanted adults in comparison to normal hearing adults [46]. These findings suggest cross-modal recruitment by vision in this group of cochlear-implanted adults. The cortical sources in auditory temporal cortex for the CI users in this study were negatively correlated with speech recognition, evidence that such cross-modal plasticity may be maladaptive in nature [46].

Another form of sensory plasticity in the context of hearing loss is intra-modal plasticity. Unlike cross-modal plasticity in which sensory deprivation in one modality may lead to recruitment of cortical resources from another modality, intra-modal plasticity refers to changes that occur within a modality-specific cortices as a result of altered (increased or decreased) sensory input [55,56]. For example, a study by Doucet and colleagues (2006) demonstrated that cochlear implanted adults with good speech perception demonstrated larger P2 cortical visual evoked potential amplitudes when compared to either normal hearing adults or adults with cochlear implants who have poor speech perception [47]. Moreover, whereas the poor performers showed diffused cortical sources in response to visual stimulation across the cortex, including auditory temporal regions (taken as evidence of cross-modal re-organization), the good performers showed preserved cortical sources in large areas of visual cortex alone. The enhanced visual P2 amplitude and cortical sources restricted to visual cortex was interpreted as evidence of intra-modal re-organization in the good cochlear implant performers, whereby deafness led to enhanced compensation in the visual modality [47]. In another study of 12 post-lingually deafened adults [39], a positive correlation was observed between presence of significant sources in visual cortex during a speech-processing task before implantation and speech perception performance 6 months after cochlear implant use. Taken together, these studies suggest that intra-modal re-organization by vision may synergistic in nature, facilitating improvements in speech perception following implantation [38,39,47,57]. Cross-modal patterns have been observed in the somatosensory modality in deaf adult populations, where somatosensory stimulation elicits auditory cortical regions [23,29,58] in long-term deaf individuals. Overall, there is a need for a deeper understanding of within-modality (intra-modal) and between modality (cross-modal) plasticity in clinical populations with hearing loss, as these changes may directly influence outcomes following audiological intervention.

## 6. Cross-Modal Plasticity in Age-Related Hearing Loss

Importantly, and perhaps surprisingly, evidence of cross-modal re-organization has been seen not just in the extreme case of long-term severe-profound deafness and in adults and children, but also in adults with lesser degrees of hearing loss [36]. For example, in a recent study by our group, cortical visual evoked potentials (CVEPs) were recorded in a group of normal hearing adults and a group of adults with early-stage, mild-moderate sloping hearing loss from 2–8 kHz [36]. Many of the adults in the hearing loss group were unaware of their hearing loss at the time of enrollment in the study, suggesting that the hearing loss was relatively recent. Results revealed that normal hearing listeners demonstrated expected cortical sources in higher-order visual and cerebellar regions to an apparent visual motion stimulus, while listeners with mild-moderate hearing loss demonstrated additional recruitment of temporal (auditory) cortical regions in response to the same visual stimulus, suggestive of cross-modal re-organization. Additionally, a negative correlation was observed between N1 visual evoked potential latency and participants’ speech perception in noise performance, suggesting that cross-modal recruitment by vision occurred in early-stage hearing loss may negatively impact speech perception outcomes for this population [36].

In addition to cross-modal sensory re-organization, there is also growing evidence of compensatory changes in cortical resource allocation in older adults and adults with early-stage, age-related, hearing loss. For example, our group [52] recorded cortical auditory evoked potentials (CAEPs) in normal hearing adults and adults with mild-moderate early stage hearing loss. While auditory stimulation elicited sources in auditory cortex (superior and middle temporal gyrus) in the normal hearing adults, the adults with early-stage hearing loss demonstrated decreased cortical sources over temporal cortex in addition to significant cortical sources in frontal and pre-frontal cortex. Given the functional role of frontal and pre-frontal areas in tasks of working memory and executive function, recruitment of frontal networks in early-onset hearing loss may reflect effortful listening consistent with previous results [59,60,61,62,63]. Taken together, it appears that hearing loss (even mild) results in diminished cortical sources in temporal regions which likely leads to an increase in listening effort and cognitive load, as evidenced by frontal recruitment observed during auditory tasks. As a means to compensate for the increased listening effort, functional cross-modal re-organization by vision and potentially somatosensation may occur. Given the strong correlation between hearing loss and cognitive decline among older adults [64,65,66], it is worth speculating that this re-allocation of functional resources (e.g., recruitment of cross-modal cortical regions and frontal cortex) during auditory tasks may negatively impact cognitive reserve in hearing impaired listeners potentially contributing to cognitive decline.

Whether or not audiological intervention has the ability to reverse maladaptive changes in cortical resource allocation and cross-modal plasticity and/or to improve cognitive function remains unknown. In a recent study by Mosnier and colleagues (2015) in a group of elderly patients [67], 81% of the adults who scored low on measures of cognition saw a significant improvement in global cognitive function within six months after cochlear implantation. Future studies should systematically examine the effects of audiological intervention on cognition, cross-modal plasticity, and changes in cortical resource allocation in clinical populations with age-related hearing loss.

## 7. Conclusions

The recurrent theme outlined in this review is that auditory deprivation results in brain re-organization, and that these brain changes may have a considerable impact on variability in clinical outcomes in patients with hearing loss. This review provides evidence of cortical cross-modal re-organization, whereby cortical auditory brain regions are recruited in deafness or in hearing loss to carry out processing in other sensory modalities such as vision, somatosensation. More studies are needed to further elucidate the clinical applications of cross-modal re-organization in patients with hearing loss. To this end, we have demonstrated several examples of cross-modal re-organization in individual subjects with hearing loss including children with cochlear implants who are excellent and average users and show differing levels of cross-modal re-organization [23] and a child with SSD received a cochlear implant which allowed reversal of cross-modal re-organization [45]. We have reviewed and compared cross-modal re-organization with intra-modal re-organization, where there are changes in cortical processing in a single sensory modality following sensory deprivation, and provided evidence that while cross-modal changes are maladaptive in nature, intra-modal changes are likely adaptive (beneficial) in nature [38,39,47,48,68,69]. Finally, we have discussed compensatory cross-modal plasticity as a response to effortful listening in early stage, age-related hearing loss [45,52]. Given the fact that hearing loss is the one of the most common chronic health condition among older adults, with its prevalence projected to increase in coming years [70], and the fact that hearing impaired adults wait over a decade on average before seeking intervention, markers of intra-modal and cross-modal plasticity may become valuable assets in the early identification and intervention process.
